# Emergence of complex structures from nonlinear interactions and noise in coevolving networks

**DOI:** 10.1038/s41598-020-72662-8

**Published:** 2020-09-24

**Authors:** Tomasz Raducha, Maxi San Miguel

**Affiliations:** 1 Institute of Experimental Physics, Faculty of Physics, University of Warsaw, Pasteura 5, 02-093 Warsaw, Poland; 2 IFISC, Institute for Cross-disciplinary Physics and Complex Systems (UIB-CSIC), Campus Universitat Illes Balears, 07122 Palma de Mallorca, Spain

**Keywords:** Complex networks, Nonlinear phenomena

## Abstract

We study the joint effect of the non-linearity of interactions and noise on coevolutionary dynamics. We choose the coevolving voter model as a prototype framework for this problem. By numerical simulations and analytical approximations we find three main phases that differ in the absolute magnetisation and the size of the largest component: a consensus phase, a coexistence phase, and a dynamical fragmentation phase. More detailed analysis reveals inner differences in these phases, allowing us to divide two of them further. In the consensus phase we can distinguish between a weak or alternating consensus and a strong consensus, in which the system remains in the same state for the whole realisation of the stochastic dynamics. In the coexistence phase we distinguish a fully-mixing phase and a structured coexistence phase, where the number of active links drops significantly due to the formation of two homogeneous communities. Our numerical observations are supported by an analytical description using a pair approximation approach and an ad-hoc calculation for the transition between the coexistence and dynamical fragmentation phases. Our work shows how simple interaction rules including the joint effect of non-linearity, noise, and coevolution lead to complex structures relevant in the description of social systems.

## Introduction

Statistical physics models of social behaviour^1^ aim at describing collective phenomena emerging from interactions. Simple Ising-type models follow a mechanistic approach, while other more complex models go beyond this concept including individual goals and incentives^2^, emotions^3^ and cognition^4^. The simple mechanistic approach is useful to identify questions that can be properly answered, isolate a mechanism of interaction and determine its consequences at the collective level, as well as to establish cause-effect relations. A prototype simple model of this kind is the voter model^5,6^, considered in the physics literature as a non-equilibrium lattice model^7^. With a binary spin-like state it is often used as a model of opinion dynamics describing the mechanism of social imitation. In spite of its simplicity, different forms and extensions of the model have been fruitful in explaining empirical observations in fairly distinct phenomena such as electoral processes^8^, stock market^9^ or online communities^10^.

There are three important elements that have been considered within the general research area of exploring consequences of empirically identified mechanisms that modify interactions in simple models: coevolution, non-linearity and noise. Coevolving or adaptive network models^11^ provide a better representation of real-world systems in comparison with static or evolving networks. Most empirical networks display both network dynamics (evolution of the network’s topology) as well as dynamics of the state of the nodes^12,13^. Therefore, coevolution can be understood as two processes—link rewiring and state dynamics—taking place at the same time and influencing each other. Moreover, a nontrivial feedback loop between these aspects renders a simple sum of effects analysed separately incomplete. Adaptive mechanisms coupling network and nodes state dynamics give rise to new phenomena absent when coevolution process is not taken into account^14–24^. Coevolution models incorporate microscopic assumptions in better agreement with empirical observations, and they also produce new macroscopic results.

Another essential feature of many real-world systems is the non-linearity associated with non-dyadic interactions. It is often assumed in network models that an interaction occurs pairwise, only between two selected vertices. From a single node point of view it means selecting one of its neighbours at random for the interaction. This leads to a linear relation between the number of neighbours in a given state and the probability of choosing one of them. However, in non-dyadic or group interactions, linearity is lost^25^. In contagion or spreading processes, the difference between these two types of interaction goes under the name of simple vs. complex contagion^26–28^. More general approaches to group interactions, based on structures of higher order than links connecting two nodes, have been recently reviewed^29^. In particular, the coevolving voter model on a certain class of hypergraphs has been considered^30^.

A third crucial empirical element in many dynamical processes on networks is noise. This is specially important in social systems where noise is inevitable^1,31^. It can manifest itself on various levels. First, people chose other people to interact with at random. The exact form of this randomness can take different forms, nevertheless the structure’s evolution is never hard-coded. But the most fundamental part of randomness lays probably within individual choices. For example, having exactly the same influence on two people’s opinions we can not be sure of the outcome. This mechanism is sometimes referred to as non-conformism^32^. It reflects the ability of agents to change state independently of the states of their neighbours. It is often a model parameter that needs to be calibrated to reproduce empirical data^8^.

In this paper we aim at exploring the joint effect of these three important aspects—the coevolution of network structure and node states, the non-linearity of interactions and the noise—on the behaviour of the system. As the framework we choose the simple voter model^5,6^. The consequences of coevolution^33–35^, noise^36–39^, and non-linearity^25^ have been already considered separately in the voter model. The joint effect of these aspects, however, turns out to be more complex than a mere superposition of the results obtained so far.

The coevolving voter model (CVM)^33^ was among the pioneers in introducing adaptive mechanisms in general. In the standard voter model, node state dynamics follows an imitation rule which is here coupled with link rewiring, introducing the coevolution. This leads to a network fragmentation transition. The effect of noise in the CVM^40^ prevents the existence of absorbing configurations so that the different phases of the system are described by dynamically active stationary states that do not collapse but are stable. These include a striking new dynamical fragmentation phase. Additionally, a fully-mixing phase is found, as could be expected for large noise levels. Nonlinear interactions have been also considered in the CVM^41,42^. Non-linearity changes the stability of fixed points in the voter model dynamics, leading to a new dynamically trapped coexistence phase. Finally, the joint effects of noise and non-linearity in the voter model have also been considered^43^. It was found that non-linearities transform a finite size transition known from the noisy voter model into a bona fide phase transition that survives in the thermodynamic limit.

In this paper we introduce a CVM in which noise and non-linearities are jointly taken into account. We obtain a full phase diagram in the three-parameter space measuring the rate of network adaptability in the coevolution dynamics, the strength of non-linearity and the noise intensity. We find three different phases: consensus, coexistence and dynamical fragmentation. The consensus phase is further divided into a strong consensus phase, with a realisation of the process staying always in a given consensus, and an alternating consensus phase. The coexistence phase can be also divided into a fully-mixing and structured coexistence. The strong consensus phase and the coexistence phase are the only ones that survive in the thermodynamic limit.

**Figure 1:**
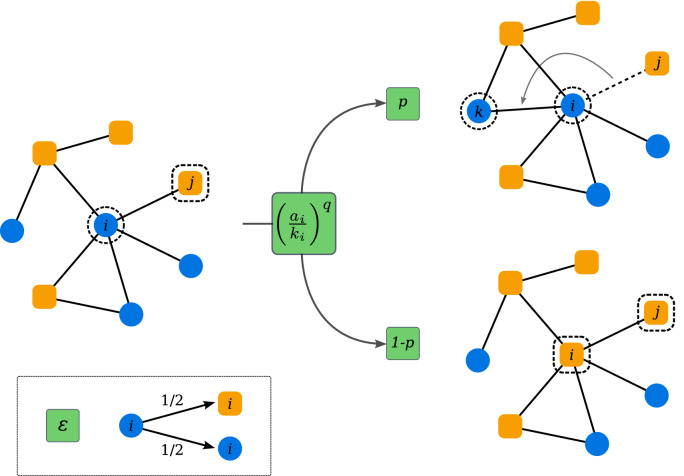
Schematic illustration of update rules in the nonlinear coevolving voter model with noise. Every node is in a state $+1$ or $-1$, indicated by orange and blue colours. The active node *i* is chosen randomly. Then with probability $({a_i}/{k_i})^q$ an interaction occurs and one of the active links (the one to the node *j*) is selected randomly. With probability *p* the link (*i*, *j*) is rewired to the link (*i*, *k*), where *k* is a random node being in the same state as *i*. With probability $1 - p$ the focal node *i* copies the state of the node *j*. At the end of the time step, regardless of what happened before, the active node draws a random state with probability $\epsilon$.

### The model

Our noisy and nonlinear CVM is defined by its time evolution over discrete time steps as follows. First a random graph is generated and every node is assigned a state $s_i \in \{-1,+1\}$ at random. (Every time something is done *at random* without specifying the probability distribution it means the distribution is uniform, i.e. probability is constant for every outcome.) In each time step a node *i* is chosen at random, we call it the active or focal node. Then, with probability $({a_i}/{k_i})^q \equiv \rho _i^q$ an interaction occurs, where $k_i$ is the degree of the focal node *i*, $a_i$ is the number of neighbours of the node *i* being in the opposite state, and *q* is the non-linearity parameter of the model. If an interaction occurs, one of the $a_i$ neighbours in a different state is chosen randomly, call it *j*. Then, with probability *p* a link rewiring is performed and with complementary probability $1-p$ a state copying. When rewiring, the node *i* cuts the connection to the node *j* and creates a new link with a randomly selected node in the same state (if there is no such node, nothing happens). When copying the state, the node *i* replicates the state of the node *j*, i.e. $s_i \rightarrow s'_i = s_j$. At the end of the time step, regardless of what happened before, the active node with probability $\epsilon$ draws a random state. Note that this is equivalent to flipping the current state with probability $\epsilon /2$. The algorithm of the model is illustrated in Fig. 1.

Our model has three parameters, namely the noise rate $\epsilon$, the plasticity *p* and the non-linearity *q*. The parameter *p* is a network plasticity parameter measuring the ratio of time scales of node dynamics and network dynamics. The non-linearity parameter *q* measures the nonlinear effect of local majorities: $q=1$ corresponds to the ordinary voter model with a mechanism of random imitation, while $q<1$ indicates a probability of imitation above random imitation and $q>1$ a probability below random imitation. The ordinary voter model corresponds to $p=\epsilon =0$ and $q=1$, while $p=\epsilon =0$ corresponds to the nonlinear voter model and $p=0$ and $q=1$ to the noisy voter model . The CVM is obtained for $\epsilon =0$ and $q=1$, the noisy CVM is obtained for $q=1$ and the nonlinear CVM for $\epsilon =0$.

Our simulations are ran from an initial random network with *N* nodes and *M* links or average degree $\mu = \sum _i k_i / N = 2M/N$, and with random initial conditions for the nodes states until a stationary state or a frozen configuration is reached (the latter being possible only for $\epsilon =0$). Note that due to the random rewiring the network’s structure remains random for every parameter configuration.

## Results

We explore the space of possible values of the parameters ($p,q, \epsilon$) by means of computer simulations and analytical approximations. In order to describe the system, we use typical order parameters, such as magnetisation $m = \sum _i s_i / N$, size of the largest network component *S*, and density of active links $\rho = \sum _i a_i / 2M$. By an active link we mean a connection between two nodes being in opposite states. All these values are usually normalised to fit the range [0, 1]. Additionally, we define a new indicator, namely community overlap (*ov*.), in order to be able to distinguish structural changes. The community overlap measures the fraction of nodes assigned to the same community by both their state and by the algorithm of community detection in the network^46^. Consequently, if a given node was assigned to the same community in both cases it increases the *ov*. by 1/*N* (where the denominator comes from normalisation). In the community detection research a notion of *overlapping communities* is common in the sense of nodes being assigned to different topological communities^44,45^. Note, that we use *community overlap* in the sense of nodes being assigned to the same community by different methods (see the precise definition further in the text).

**Table 1:** Average values of the order parameters in different phases. The border between phases A and B is given by $|m|=0.5$, between phases B1 and B2 is given by $ov.=0.75$ which is approximated by $\rho =0.1$, the border between phases B and C is given by $S=0.75$

Order parameter	Phase			
	A	B1	B2	C
$\langle |m| \rangle$	$\lesssim 1$	$\gtrsim 0$	$\gtrsim 0$	$\gtrsim 0$
$\langle S \rangle$	1	1	$\lesssim 1$	$\gtrsim 0.5$
$\langle ov. \rangle$	–	$\gtrsim 0.5$	$\lesssim 1$	1
$\langle \rho \rangle$	$\gtrsim 0$	Finite	$<0.1$	$\gtrsim 0$

**Figure 2:**
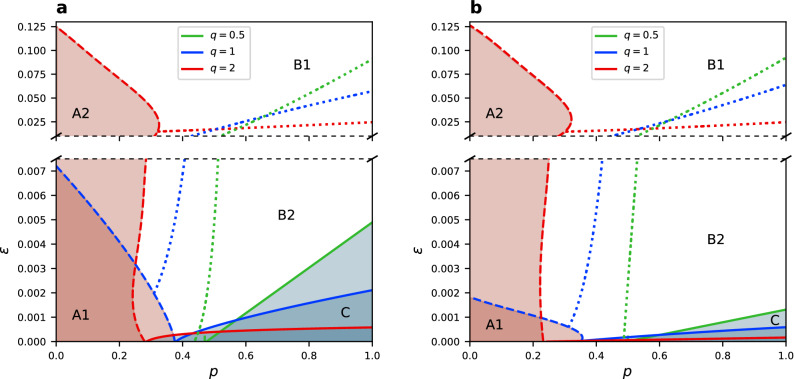
Phase diagram in the *p*-$\epsilon$ space for (**a**) $N=250$ and (**b**) $N=1000$ for $\mu =4$ and different values of *q* (indicated by colour). Results based on simulations averaged over 500 realizations. The red area represents the phase A, the white one phase B, and the blue one phase C. The border between phases A and B is a line defined by the medium value of the average (over time in one stochastic realization) absolute magnetization $\langle |m| \rangle =0.5$ (dashed lines). The border between phases B and C is a line defined by the medium size of the largest component $\langle S \rangle =0.75$ (solid lines). The border between phases B1 and B2 (dotted lines) is approximated by $\langle \rho \rangle =0.1$ (see Table 1).

The difference between phases A1 and A2 can be observed in the dynamical behaviour of the magnetisation and on the system size scaling (Figs. 2 and  7). See Supplementary Information for examples of stationary states for each phase.

### Phase diagram

We numerically study the *p*-$\epsilon$ phase diagram for three different values of the *q* parameter—the sublinear case $q=0.5$, the ordinary linear case $q=1$, and the superlinear case $q=2$. These phase diagrams are shown in Fig. 2 for two different network sizes. Changes in the average degree $\mu$ can influence the position of phase transitions and increase the active link density $\rho$ (see “Methods” section), however they do not change the general behaviour of the system. Obviously, for any finite amount of noise in the system a frozen configuration does not exist, and any phase is described by a characteristic dynamical stationary state. We can distinguish three general phases in the model. Phase A, indicated by the red area in the figure, is a consensus phase. In this range of parameters the system stays in a consensus state for most of the time, i.e. magnetisation is close to $\pm 1$ and the network is connected having a single large component $S=1$ and a small number of active links $\rho \gtrsim 0$ (see Supplementary File 1). If we increase the noise rate $\epsilon$ or the plasticity *p* sufficiently, we obtain phase B indicated by the white area in Fig. 2, and referred to as a fully-mixing phase in previous work for the noisy CVM^40^. In this phase the magnetisation drops to zero, $m=0$, hence there is no consensus in the system any more. In addition, the network stays connected most of the time, $S\lesssim 1$ . We refer to this phase as a coexistence phase. As we will see, phase B is not homogeneous in its whole range of parameters and it can be either fully-mixing (phase B1, see Supplementary File 2) or structured (phase B2, see Supplementary File 3), what is indicated by different values of the density of active links $\rho$ and community overlap *ov*. Finally, for values of the rewiring probability above the critical point $p_c$ of the nonlinear CVM^41^, and relatively small noise rates, phase C arises. It is marked by the blue area in the figure. In this region we find dynamical fragmentation—the network consists of two separate components with nodes in each one being in opposite states, so that $m\gtrsim 0$, $S\gtrsim 0.5$ and $\rho =0$. It is possible, however, that the two network components get connected intermittently in the stationary state due to noise and random rewiring, creating again a single component network with $m\gtrsim 0$ and a small number of active links $\rho \gtrsim 0$ (see Supplementary File 4). Phase C can be described as dynamical switching between these two arrangements. Values of all analysed quantities for every phase are summarised in Table 1. Videos presenting exemplary dynamics of stationary states for all phases are provided in Supplementary Information.

**Figure 3:**
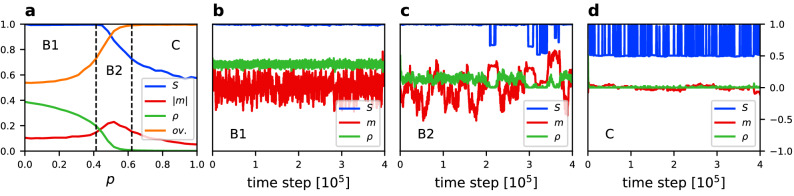
(**a**) Average value of the size of the largest component $\langle S \rangle$, absolute magnetization $\langle |m| \rangle$, density of active links $\langle \rho \rangle$, and community overlap $\langle ov. \rangle$ vs. rewiring probability *p* for $q=0.5$, $\epsilon =0.001$, $N=250$, and $\mu =4$. Results averaged over 500 simulation runs. Borders between phases are indicated by dashed lines according to the Table 1. For every phase a trajectory of *S*, *m* and $\rho$ is given for the same parameters values and (**b**) $p=0.1$, (**c**) $p=0.46$, (**d**) $p=0.8$.

**Figure 4:**
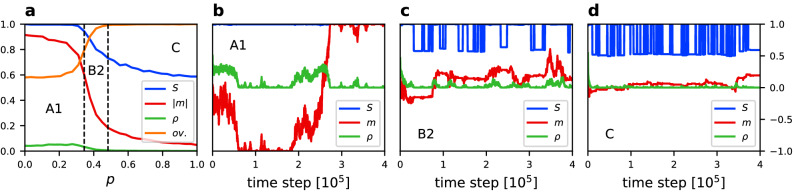
(**a**) Average value of the size of the largest component $\langle S \rangle$, absolute magnetization $\langle |m| \rangle$, density of active links $\langle \rho \rangle$, and community overlap $\langle ov. \rangle$ vs. rewiring probability *p* for $q=1$, $\epsilon =0.0005$, $N=250$, and $\mu =4$. Results averaged over 500 simulation runs. Borders between phases are indicated by dashed lines according to Table 1. For every phase a trajectory of *S*, *m* and $\rho$ is given for the same parameters values and (**b**) $p=0.1$, (**c**) $p=0.38$, (**d**) $p=0.6$.

**Figure 5:**
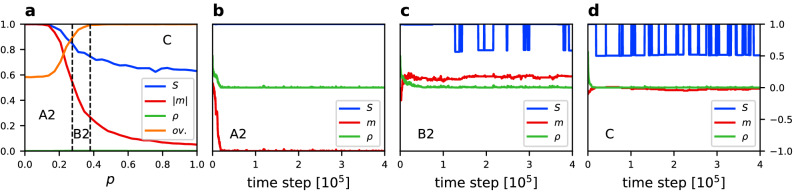
(**a**) Average value of the size of the largest component $\langle S \rangle$, absolute magnetization $\langle |m| \rangle$, density of active links $\langle \rho \rangle$, and community overlap $\langle ov. \rangle$ vs. rewiring probability *p* for $q=2$, $\epsilon =0.0002$, $N=250$, and $\mu =4$. Results averaged over 500 simulation runs. Borders between phases are indicated by dashed lines according to Table 1. For every phase a trajectory of *S*, *m* and $\rho$ is given for the same parameters values and (**b**) $p=0.2$, (**c**) $p=0.32$, (**d**) $p=0.9$.

**Figure 6:**
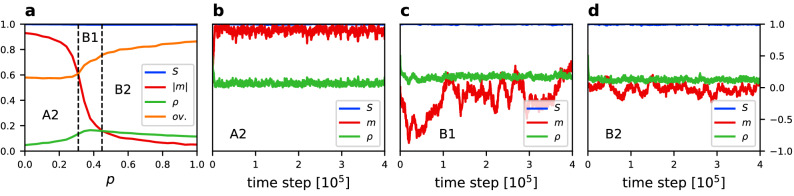
(**a**) Average value of the size of the largest component $\langle S \rangle$, absolute magnetization $\langle |m| \rangle$, density of active links $\langle \rho \rangle$, and community overlap $\langle ov. \rangle$ vs. rewiring probability *p* for $q=2$, $\epsilon =0.03$, $N=250$, and $\mu =4$. Results averaged over 500 simulation runs. Borders between phases are indicated by dashed lines according to Table 1. For every phase a trajectory of *S*, *m* and $\rho$ is given for the same parameters values and (**b**) $p=0.1$, (**c**) $p=0.38$, (**d**) $p=0.9$.

For the linear case ($q=1$) phases A and C exist only for a finite size of the network, and the size of these phases in the parameter space decreases with growing number of nodes. For the sublinear scenario $q<1$, we can see in Fig. 2 that the same holds for phase C, while phase A does not exist at all. The only point where the average absolute magnetization slightly raises is at $p_c$ and for $\epsilon \approx 0$, but its maximal value is only about 0.3. This raise is due to higher fluctuations close to the transition point. On the other hand, phase C prevails for even twice larger noise rate than in the linear scenario.

In the superlinear case ($q=2$) phase C is much smaller in parameter space and disappears faster with growing system size. But phase A prevails for much larger noise than in the linear case $q=1$. We observe phase A even for $\epsilon$ larger by almost two orders of magnitude. Additionally, the system size scaling is different for the superlinear scenario. Indeed, non-linearity has significant influence on the nature of phase A. For $q<1$ it does not exist, for $q=1$ it exists only in finite networks, and for $q>1$ phase A persists in the thermodynamic limit.

### A closer look at the phases

In order to better understand the behaviour of the system and the differences between phases we analyse horizontal cross-sections of Fig. 2 and single-run trajectories which are presented in Figs. 3, 4, 5 and 6 for the linear, sublinear and superlinear cases. In panel (a) of each of them, a phase diagram with respect to rewiring probability *p* and for a particular value of $\epsilon$ is presented. Values of the noise rate are chosen in such a way that allows to show three phases in one panel. For an horizontal cross-section of the full phase diagram it is difficult to capture all phases. Therefore, areas of the middle phase can be narrow, but still different values of the order parameters can be distinguished. Panels (b)–(d) show typical time traces of the order parameters in different phases.

For the sublinear case ($q=0.5$) we can see the differences between phase B1 (fully-mixing, Fig. 3b) and phase B2 (structured, Fig. 3c). Phases B1 and B2 have zero average absolute magnetisation $\langle |m| \rangle \approx 0$, but we can distinguish a region with high density of active links (B1) and small density of active links (B2). Phase C also has a low density of active links, but the largest network component switches from $S=1$ to $S=0.5$, giving the average value $\langle S \rangle \approx 0.5$, whereas phase B2 on the average stays connected.

Results for the linear case ($q=1$) are shown in Fig. 4. Phase A is characterised by a magnetisation which tends to stay at one of the consensus states, but it can switch from $-1$ to $+1$, or the other way around, during the time evolution. Therefore, $\langle |m| \rangle \approx 1$ but $\langle m \rangle =0$. To distinguish it from the superlinear case where $\langle m \rangle \approx \pm 1$ we call this phase A1. We also observe that for $q=1$ in phase B2 (Fig. 4c) the network can fragment close to the transition line, however it remains a single component network most of the time.

Figures 5 and 6 correspond, respectively, to small and large noise rates in the superlinear case ($q=2$). In this scenario the consensus phase A prevails for much larger noise rate. In panels (b) of Figs. 5 and 6 we can see how the system behaves in phase A for $q=2$. It quickly reaches a consensus state for either $m=1$ or $m=-1$ and remains at this value of magnetisation. Therefore, $\langle |m| \rangle \approx 1$ and in contrast to the linear case in a time average also $\langle m \rangle \approx \pm 1$. To account for this difference we call the consensus phase A2 for $q>1$ .

**Figure 7:**
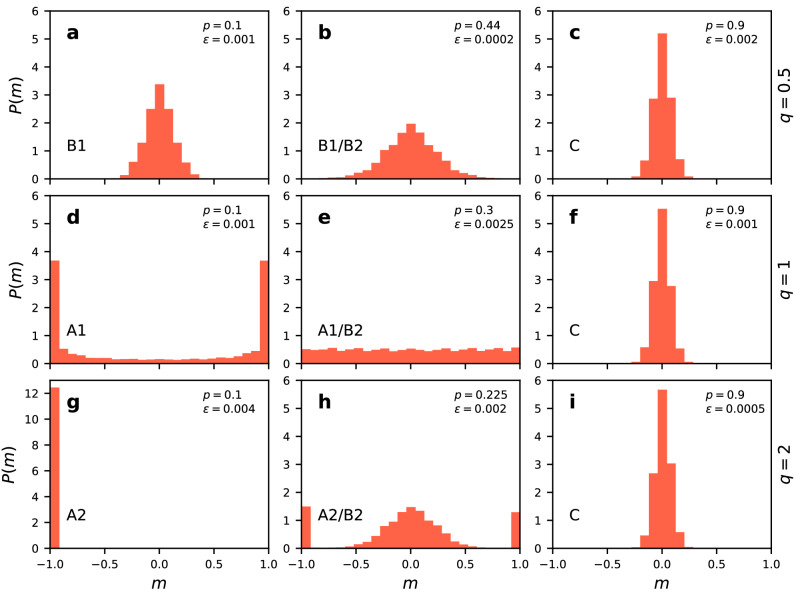
Probability distribution of the magnetisation *m* for $N=250$ and $\mu =4$ averaged over $10^7$ MC steps after thermalization. Results for (**a**–**c**) $q=0.5$, (**d**–**f**) $q=1$, and (**g**–**j**) $q=2$. Values of the rewiring probability *p*, noise rate $\epsilon$ and the phase indication are given in the panels.

The difference between the consensus phase A1 and A2 is also clearly visible from the probability distribution of the magnetisation in a given realisation of the dynamical process (Fig. 7). In the linear case there is a bimodal distribution for the magnetisation with two equal peaks at values $+1$ and $-1$ (Fig. 7d), while for the superlinear case there is a single peak for a value of the magnetisation at either of the boundary values $+1$ or $-1$, depending on the run (Fig. 7g). For $q=2$, once the consensus is reached the system stays there with minor fluctuations (phase A2), while for $q = 1$ the system goes back and forth between opposite consensus states (phase A1). Furthermore, phase A2 is robust against finite-size fluctuations, while phase A1 disappears in the thermodynamic limit^40^ (see Fig. 2).

The distribution of the magnetisation gives additional insights on the phase diagram: The fact that phase A does not exist in the sublinear case ($q < 1$), is reflected in a distribution with a single peak at 0 for all values of $\epsilon$ (Fig. 7a–c). However, the variance of the distribution takes its maximal value for noise going to zero and $p=p_c$, i.e. close to the transition point between coexistence and fragmentation phase in the nonlinear CVM^41^. A different form of the transition between phases A and B for the linear and superlinear case is also observed. For $q=1$ there is a flat distribution at the transition point (Fig. 7e) , while a trimodal distribution is found for $q=2$ (Fig. 7h). A trimodal magnetization distribution was reported before in the noisy voter model on a static network^43^, but only for non-linearity parameter equal 5 or larger. With coevolution, trimodality here is obtained already for $q=2$.

**Figure 8:**
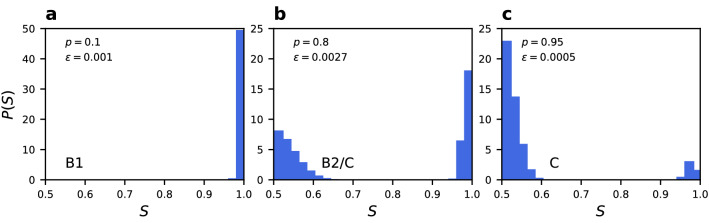
Probability distribution of the size of the largest component *S* for $N=250$, $\mu =4$, and $q=0.5$ averaged over $10^7$ MC steps after thermalization. Results for (**a**) phase B1, (**b**) close to the transition line and (**c**) phase C. Values of the rewiring probability *p* and noise rate $\epsilon$ are given in the panels.

Phase C can be defined in terms of the size of the largest component. In phases A and B it is equal to the size of the whole network ($S=1$), while the phase C is characterised by a dynamical fragmentation into two components of similar size and opposite state. Due to noise expressed in random changes of nodes states and rewiring the components are constantly being reconnected and disconnected. It can be examined looking at the trajectory or at the probability distribution of the size of the largest component, which is presented in Fig. 8.

### Community structure

**Figure 9:**
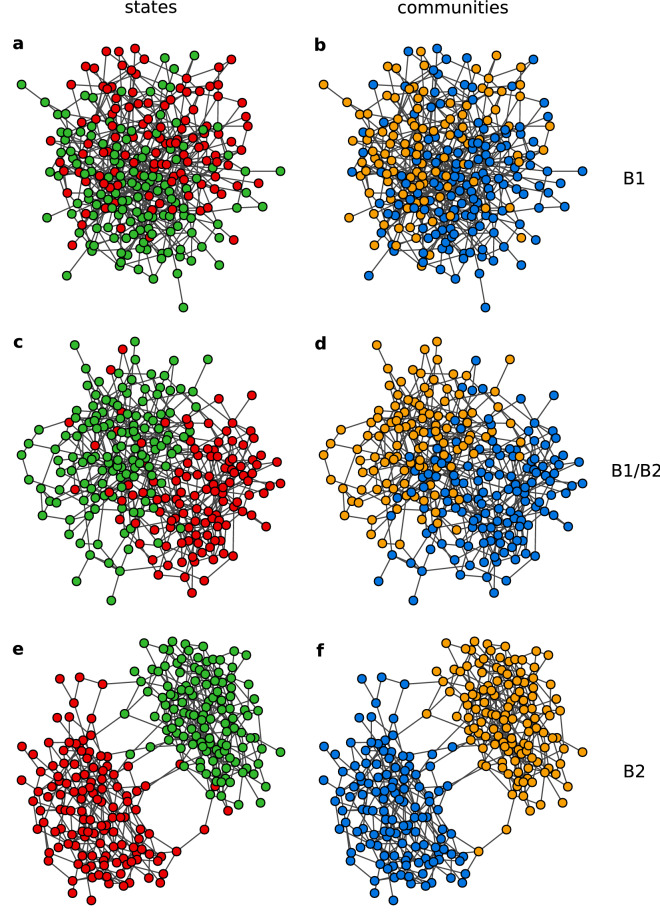
Examples of the network topology and node states in the stationary state for (**a**,**b**) phase B1 at $p=0.9$, $\epsilon =0.004$, (**c**,**d**) the transition line between B1 and B2 at $p=0.5$, $\epsilon =0.025$, and (**e**,**f**) phase B2 at $p=0.5$, $\epsilon =0.1$. In all cases $N=250$, $M=500$ and $q=2$. State $+1$ is indicated by green colour and $-1$ by red (**a**,**c**,**e**). Communities found by the community detection algorithm^46^ are coloured blue and orange (**b**,**d**,**f**). In the B1 communities are not associated with the states of the nodes, so that the overlap between (**a**) and (**b**) is 0.54. On the transition line states start to rearrange into communities giving overlap equal 0.77 for (**c**) and (**d**). In phase B2 the communities defined by the state of the nodes are well overlapping with the structural communities giving overlap of 0.97 for (**e**) and (**f**).

Phase B is generally defined by zero average magnetisation, also zero average absolute magnetisation, and by the existence of one large network component. Nonetheless, this description leaves room for different possible configurations. Analysis of the trajectories showed that the density of active links can vary within phase B, but the question is weather this is a sign of a topological change. In the linear case ($q=1$) only the fully-mixing phase was reported^40^, with nodes of states $+1$ and $-1$ well mixed inside a random graph. We refer to this configuration as phase B1. On the other hand, we can satisfy conditions for the phase B having two evident communities, highly connected internally and of opposite states, with only a few links bridging them. There is still zero magnetisation and one large network component in such configuration characterised by a small number of active links. We call this phase B2. The difference between phases B1 and B2 is clearly seen in Fig. 9.

Although the difference between phases B1 and B2 can be seen in the density of active links, a closer look at Fig. 9 suggests that phase B2 has well defined topological communities. Therefore, we propose an alternative quantitative measure for the difference between phases B1 and B2, the overlap between state communities—defined by the state of the nodes—and structural communities found by a community detection algorithm. We use a classical algorithm from^46^, but the result does not differ much when using other algorithms. For a wider discussion of community detection methods see^44^. Each node is assigned to the state community by its state and to a structural community by the algorithm’s result. The relative overlap between these two communities is a new quantitative indicator of the phase of the system. Note, that one has to consider all possible permutations of the assignment in order to precisely identify the overlap—we have to take the maximum overlap from two possible community assignments. If we have structural communities *a* and *b*, we can associate community *a* with the state $+1$ and community *b* with $-1$, or the other way around. Therefore, in a perfect overlap with wrong assignment one can get zero overlap. Trying both possibilities and taking maximum solves this issue. For a random assignment or no community structure the overlap will be close to 0.5. This is the situation in phase B1. For phase B2 the overlap will be close to 1. This means that our dynamical coevolving model generates clear topological communities emerging from local interactions involving only state of nodes. This result may potentially explain process of formation of communities in social networks, where such structures are especially common^47^. Different approaches to identifying the overlap between state communities and structural communities are possible. For instance, a common method is analysing all possible pairs of nodes and counting those in which both nodes are assigned the same community. Then, the result can be reduced to a single number by e.g. the Jaccard index. Such overlap will have a different range, however our simulations showed that it perfectly correlates with the overlap indicator defined here.

### Identifying phase boundaries

So far, we gave a description of different phases with different qualitative behaviour. Transition lines between these different types of behaviour are not clearly or unambiguously defined because every analysed phase indicator changes value significantly across the phase diagram. We do not focus on properties of phase transitions in this work, but rather on the properties of different emerging structures. Therefore we follow here a simple and pragmatic way to identify boundaries between different phases, employing previous approaches^40^. These boundaries have to be understood as an arbitrary way to deal with crossover system behaviour.

Each of the order parameters or phase indicators analysed has a continuous range of values (for a large *N*) and different phases are described by the extreme values of these quantities, allowed in this range. Therefore, a straightforward way of dividing the phase diagram into separate phases is to use the middle value, i.e. the value in the middle between the maximum and minimum of a given range. For example, the absolute magnetisation is defined in the range [0, 1], taking a value close to 1 in phase A and a value close to zero in phase B. Hence, we identify the border between the two phases with $\langle |m| \rangle =0.5$.

Likewise, the size of the normalised largest network component takes values in the range [0.5, 1]. In phases A and B there is a single component network ($S=1$), while phase C is characterised by the dynamical fragmentation into two components of similar size and opposite state, so that $S=0.5$. In this phase, due to noise expressed in random changes of nodes states and rewiring, the components are constantly being reconnected and disconnected. We then identify^40^ the border between phase B and phase C by the middle value $S=0.75$. This is a line at which the network is half of the time fragmented and half of the time contains only one big component. This phase boundary can also be obtained from the probability distribution of the size of the largest component (Fig. 8). At the transition line (panel b) two peaks have the same area.

Finally the boundary between phases B1 and B2 can be identified in terms of the the overlap (*ov*.), which takes values in the range [0.5, 1]. There is a transition from values around 0.5 in B1, up to 1 in B2, and so we define the transition line at $ov.=0.75$. However, this parameter is computationally very demanding. Alternatively, we can approximate the identification of the transition line by a small value of the density of active links which we arbitrarily fix at $\rho =0.1.$

### Analytical predictions

**Figure 10:**
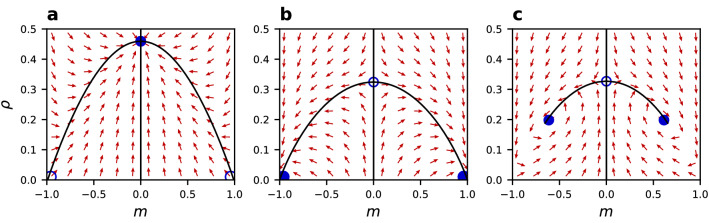
Flow diagram of the system dynamics in the *m*-$\rho$ space for $\mu =8$, $p=0.1$ and (**a**) $q=0.5$, $\epsilon =0$, (**b**) $q=2$, $\epsilon =0$, (c) $q=2$, $\epsilon =0.1$. Arrows represent the dynamical direction of the system according to the pair approximation dynamics (Eq. 3). Fixed points are represented by full circles (stable) and empty circles (unstable). Note how non-linearity and noise can change the stability and position of the fixed points.

**Figure 11:**
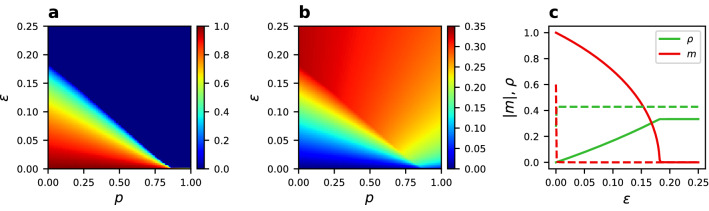
Numerical solution of the pair approximation (Eq. 3). (**a**) Absolute magnetization |*m*| and (**b**) density of active links $\rho$ for $q=2$ and $\mu =8$ in the *p*-$\epsilon$ phase diagram showing the existence of phase A2 in the thermodynamic limit. (**c**) Absolute magnetization and density of active links vs. noise rate for the static case ($p=0$) and $\mu =8$. A continuous transition is present for $q=2$ (solid lines), in contrast to the case of $q=1$ (dashed lines), where consensus can only be obtained for $\epsilon =0$.

The magnetisation *m* and the density of active links $\rho$ obey Eq. 3 describing the dynamics of the system, as derived in the “Methods” section. Several fixed points $(m^*,\rho ^*)$ of these ordinary differential equations can be found depending on parameter values. However, not all of them are stable, therefore not all of them are observed in numerical simulations. To analyse the stability of these fixed points, we consider flow diagrams of the dynamics in Fig. 10. Note, that from panel (a) to (b) only the value of *q* changes, emphasising the difference between sublinear and superlinear cases, while from panel (b) to (c) only the value of $\epsilon$ changes, emphasising the noise effect in the superlinear case. A change in the non-linearity parameter *q* can reverse the stability of fixed points when going over the boundary value of 1. A change in the noise rate $\epsilon$ can additionally shift the position of fixed points allowing for fixed points different than $m=-1,0,1$. Since the analytical description is derived in the thermodynamic limit, we don’t observe stable fixed points at non-zero magnetisation for $q \le 1$. This finding is consistent with the scaling behaviour of numerical results indicating existence only of phase B in the limit of a large number of nodes *N*. For the superlinear case, although the fixed points are placed at the same values of the magnetisation $m=-1,0,1$ (for $p<p_c$, $\epsilon <\epsilon _c$), their stability is inverted—now only the solutions of $|m|=1$ are stable, corresponding to phase A2. This is clearly seen in the analytical prediction for the phase diagram in Fig. 11. These results, obtained in the thermodynamic limit, indicate that phase A2 should be observed for any *N* when $q=2$, which is in agreement with our numerical results in Fig. 2. Separate mean-field prediction of the disappearance of phase A1 in the thermodynamic limit was given by Diakonova et. al.^40^ for the linear CVM with noise. Additionally, phase C is not obtained in the thermodynamic limit.

The non-linear noisy voter model in a fixed network ($p=0$) has been thoroughly analytically studied^43^, showing that $q=1$ is a bordering value between a unimodal and bimodal distribution of the magnetization *m*. In other words, it is a transition line between existence and nonexistence of phase A. The agreement of our results with previous studies can be seen when analyzing the extreme value of $p=0$ in the phase diagrams (Fig. 11a,b). It is also separately presented in the Fig. 11c. The transition to phase A, characterized by nonzero absolute magnetization |*m*|, exists for finite values of $\epsilon$ only when $q>1$. In the “Methods” section we derive a formula for the critical value of the noise rate $\epsilon _{c}(p=0)$ at which the system looses the consensus state, that is, the transition form phase A to phase B (Eq. 7). This result is in agreement with the numerical solution of Eq. 3 presented in Fig. 11c, giving a critical value of the density of active links $\rho _c=\frac{1}{3} \approx 0.33$ and $\epsilon _{c}=\frac{2}{11}\approx 0.18$ for the parameters values used in the figure. The analytical solution from Eq. 7 also predicts disappearance of the transition at $q=1$. In the reference^43^ a similar prediction of the critical noise rate was given for a complete graph: $\epsilon '_c(p=0) = 2^{-q}(q-1)$ which would give for the case of Fig. 11c $\epsilon '_c = 0.25$. Therefore, a complete graph gives only a first approximation to the value found here.

There has been no previous attempt of an analytical approximation describing the transition from phase B to the dynamical fragmentation phase C already found in the noisy linear CVM. We propose here a simple description of this phenomena. Having two separate, but internally homogeneous network components (clusters) with nodes in opposite states, the only way of connecting them is by a random change of node’s state and link rewiring to the second component. The probability of the first event is independent of *q* and is simply given by $\epsilon /2$. When the node changing state is selected as the active node the probability of an interaction is $\rho _i^q$. Since $\rho _i \in [0,1]$, for smaller *q* the probability of an interaction is higher, except for boundary cases with $\rho _i=0,1$. To reconnect the two clusters, rewiring must occur, but this happens always with probability *p*, despite the value of *q*. Therefore, for a single node in a state opposite to the whole cluster, the probability of connecting to the other cluster is constant (since $\rho _i=1$). However, once the two clusters are connected, the higher probability of an interaction for lower *q* means a higher probability of rewiring causing fragmentation again. Consequently, we expect phase C to persist for larger noise when *q* is smaller. More detailed description of this process is given in the “Methods” section, where we derive the following formula for the transition line:

1 $$\begin{aligned} \epsilon _S (p) = \frac{4}{N} \left( \frac{1}{\mu } \right) ^{2q} (2^q + 2) p . \end{aligned}$$

**Figure 12:**
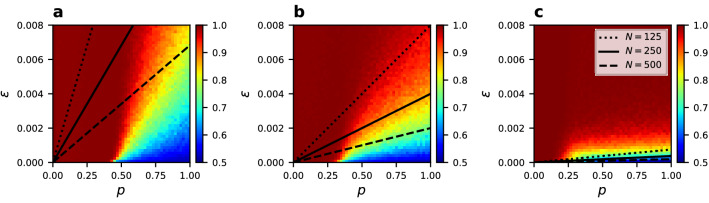
Analytical prediction of the transition line between phases B and C, according to Eq. 1, compared with simulation results for $N=250$, $\mu =4$, and (**a**) $q=0.5$, (**b**) $q=1$, (**c**) $q=2$. The analytical prediction is indicated by a solid black line. Scaling with the network size (dotted and dashed lines) and dependence on the non-linearity parameter *q* give trends consistent with simulations. Numerical results are averaged over 500 realizations.

Based on this approximation we predict phase C to fade with growing non-linearity parameter *q* or with growing system size *N*, as shown in Fig. 12. Both predictions are consistent with our numerical results.

## Discussion

In this paper we analysed the nonlinear coevolving voter model with noise. Depending on the values of the three main parameters—the rewiring probability *p*, the noise intensity $\epsilon$, and the non-linearity parameter *q*—we observed three distinct phases: a consensus phase A, a coexistence phase B, and a dynamical fragmentation phase C. We observed, however, significant internal differences within phases A and B. The first one can be further divided into phase A1 and A2. Phase A1, for $q = 1$, is a consensus phase with absolute magnetization equal 1 on average, but real magnetization switching between $-1$ and $+1$ states, giving rise to a bimodal magnetization distribution within one realization of the stochastic dynamics. In phase A2, observed for $q>1$, there is a stable consensus, i.e. global magnetization states $-1$ and $+1$ are stable. Consequently, during one realization of the stochastic dynamics the system remains in a given consensus state, producing a unimodal magnetization distribution with a peak at the maximal (minimal) magnetization. Additionally, phases A1 and A2 have different system size scaling—phase A1 disappears in the thermodynamic limit, while phase A2 is stable against finite size fluctuations. Finally, phase A does not exist for $q<1$.

Phase B can be similarly divided into phases B1 and B2. Phase B1 is a fully-mixing phase with random network structure and random states of the nodes, giving zero magnetization. But for larger *p* and low noise intensity we observe phase B2, which has the same vanishing average magnetization, but a different network structure. In the structured phase B2 one can easily distinguish two communities of opposite states connected by just a few inter-community links. This structural difference is confirmed by community detection algorithms.

Phase C is associated with a dynamical fragmentation of the network – two components in opposite magnetization states are being constantly connected and disconnected. We derive an analytical description of this behaviour and an approximated value for the transition line between phase B2 and phase C.

The only phases surviving in the thermodynamic limit are phases A2, B1 and B2. The transition line form phase A2 to phase B is largely independent of finite size fluctuations. We have also presented an analytical pair approximation able to describe these findings and the main features of phases A and B in the thermodynamic limit.

Our work fills a gap in the studies of the CVM. It provides a binding bridge between studies on the CVM with noise^40^ and studies on the nonlinear CVM^41^. It reduces to the nonlinear noisy voter model^43^ and the ordinary CVM^33^ for a proper configuration of parameters values. We obtain full consistency with those limiting cases and we explore new parameter domains. Our work brings the analysis of the voter model to a greater complexity by taking into account the joint effect of noise, coevolution and non-linearity which turns out not to be a mere superposition of them. It may provide a tool for the evaluation of the relevance of different mechanisms in the description of opinion dynamics, but can be also a reference point in the study of coevolving network models. We also show how nonlinear vs linear interactions can change the stability of a consensus state in the network and how topological communities can arise from non-topological interactions. These results are of relevant value in the description of social networks.

## Methods

### Pair approximation

We use the same approach as used for the nonlinear coevolving voter model^41^ to describe the dynamics of magnetization *m* and the density of active links $\rho$. Given the network homogeneity due to the random rewiring, we assume each node to have the same average degree $\mu = 2M/N$. Let us denote by $n_+ = (1+m) /2$ and $n_- =(1-m)/2$ the fraction of nodes in the state $+1$ and $-1$ respectively. Then, when we pick a node in the state $\pm 1$ as the active node, the probability of choosing a neighbour in the opposite state is given by ${\rho }/{2n_{\pm }}$. In other words, $\frac{\rho }{2n_{\pm }}$ gives the density of active links $\rho _i$ for a node *i* being in the state $s_i = \pm 1$. Therefore, the probability of an interaction is given by $\rho _i^q = ({\rho }/{2n_{\pm }})^q \equiv n_q^{\pm }$. When *q* takes integer values this can be also interpreted as the probability of choosing a neighbour in the opposite state *q* times. Hence, when an interaction occurs with this probability, we can make the approximation that there is at least *q* neighbours in the opposite state and for the rest of them the probability of being in a different state than the focal node is ${\rho }/{2n_{\pm }}$. All together this implies that $a_i \approx q + (\mu - q) \frac{\rho }{2n_{\pm }}$. This is a rough estimate, more precise one could be obtained using Bayes’ theorem, but it doesn’t display a significant difference in the results.

To approximate the evolution of the density of active links $\rho$ we must estimate the contributions of different events that can result in a change of $\rho$. These events are: (i) rewiring, followed by a change of state through noise, (ii) rewiring, without a change of the node’s state due to noise, (iii) changing the state of the node through state copying with no further change due to noise, and (iv) changing the state of the node only as a result of noise, with no previous state copying or rewiring. Let $\delta _{\pm }$ be the change in the total number of active links given that a node *i* such that $s_i =\pm 1$ changed state. The total change in the number of active links in the four possible events above is: (i) $1 + \delta _{\pm }$, (ii) $-1$, and for events (iii) and (iv) just $\delta _{\pm }$. Magnetization changes only when the state of the node is changed via copying or as a result of noise in three possible scenarios: state copying with no noise effect, link rewiring followed by noise effect, and no interaction—neither state copying nor link rewiring—but noise acting alone. When the focal node having a state $s_i =\pm 1$ changes its state, the total change in the magnetization is $\mp 2$. Hence, in the thermodynamic limit we have:

2 $$\begin{aligned} \begin{aligned} \frac{dm}{dt} =\,&2 (1-p)(1-\frac{\epsilon }{2}) (n_- n_q^- - n_+ n_q^+) + 2 p \frac{\epsilon }{2} (n_- n_q^- - n_+ n_q^+) + 2 \frac{\epsilon }{2} \left[ n_- (1- n_q^-) - n_+ (1- n_q^+)\right] , \\ \frac{d\rho }{dt} =\,&\frac{2}{\mu } \bigg \{ p \frac{\epsilon }{2} \left[ n_+ n_q^+ (1+\delta _+) + n_- n_q^- (1+\delta _-)\right] - p (1-\frac{\epsilon }{2}) (n_+ n_q^+ + n_- n_q^- ) \\&\,+ (1-p) (1-\frac{\epsilon }{2}) (n_+ n_q^+ \delta _+ + n_- n_q^- \delta _-) + \frac{\epsilon }{2} \left[ n_+ (1- n_q^+) \delta _+ + n_- (1- n_q^-) \delta _- \right] \bigg \} , \end{aligned} \end{aligned}$$

With a few simple algebraic transformations these equations can be rewritten as:

3 $$\begin{aligned} \begin{aligned}{}&\frac{dm}{dt} = 2 (1-p)(1-\epsilon ) (n_- n_q^- - n_+ n_q^+) + \epsilon (n_- - n_+) , \\&\frac{d\rho }{dt} = \frac{2}{\mu } \left[ (1-p)(1-\epsilon ) (n_+ n_q^+ \delta _+ + n_- n_q^- \delta _-) - p(n_+ n_q^+ + n_- n_q^- ) + \frac{\epsilon }{2} (n_+ \delta _+ + n_- \delta _-) \right] . \end{aligned} \end{aligned}$$

When node *i* changes state, all its $a_i$ active links become inactive and all other $\mu - a_i$ inactive links become active, therefore the total change in the number of active links is $\delta _{\pm } = \mu - 2a_i$. Using the previous approximation for $a_i$ we can write $\delta _{\pm } = \mu - 2q - 2(\mu - q) \frac{\rho }{2n_{\pm }}$.

The simplest stationary solution (fixed point) of Eq. 3 is given by taking the magnetization $m=0$, which leads to an equation for the stationary value of $\rho$:

4 $$\begin{aligned} - \rho ^{q+1} 2 (\mu -q) (1-p) (1-\epsilon ) + \rho ^q [(1-p)(1-\epsilon )(\mu -2q) -p ] - \rho \epsilon (\mu - q) + \frac{\epsilon }{2} (\mu - 2q) = 0 . \end{aligned}$$

A fixed point solution with $m = \pm 1$ does not exist for any finite noise rate $\epsilon$, whilst for $\epsilon =0$ a stationary solution is $\rho = 0$. Setting the noise rate to zero together with $m=0$ we obtain the stationary solution of the nonlinear CVM^41^:

5 $$\begin{aligned} \rho ^*(\epsilon =0) = \frac{(1-p)(\mu - 2q) - p}{2 (1-p)(\mu -q)} . \end{aligned}$$

For $\epsilon =0$ and $q=1$ we recover the solution of the standard CVM^33^:

6 $$\begin{aligned} \rho ^*(\epsilon =0,q=1) = \frac{(1-p)(\mu -1) - 1}{2 (1-p)(\mu -1)} . \end{aligned}$$

To compare our results with the nonlinear noisy voter model on static networks we analyse our approximation for the particular case $p=0$. Putting $\rho = \rho _c (1-m^2)$ in the first of Eq. 3 and performing a stability analysis of the fixed point solution $m=0$ we can find a critical noise value

7 $$\begin{aligned} \epsilon _{c}(p=0) = \frac{2 \rho _c^q (q-1)}{2 \rho _c^q (q-1) + 1} , \end{aligned}$$

which depends on the critical value of the density of active links $\rho _c$. The latter can be obtained from Eq. 4 as $\rho _c = \frac{\mu - 2q}{2\mu - 2q}$. This formula is shown to be in full agreement with numerical solutions of Eq. 3 shown in Fig. 11c.

### Phase C: finite size scaling

In order to describe the behaviour of the dynamical fragmentation phase we first look for an approximation for the probabilities of reconnecting two separate clusters and of disconnecting two clusters sharing at most two links. In this approach we omit events of probability proportional to $(1/N)^3$ and to $\epsilon ^2$, or of higher order.

Imagine two separate and internally homogeneous components of opposite states, as it happens in phase C. The simplest way of connecting them under the rules of the nonlinear noisy CVM involves two steps. First, one of the nodes, call it *i*, must change it’s state. This is possible only due to noise and occurs with probability $\epsilon /2$. Second, node *i* that has changed its state must rewire one of its links to a node in the opposite cluster, having the same state. This can happen with probability $p \rho _i^q / N$, because we need to select this particular node as the active node (1/*N*), an interaction has to occur ($\rho _i^q$), and rewiring must be performed (*p*). Note, that since node *i* is the only node in a different state than its cluster $\rho _i^q = 1$. Finally, it gives the probability of reconnecting two components equal:

8 $$\begin{aligned} P_r = \frac{\epsilon }{2} \frac{p}{N}. \end{aligned}$$

Approaching the transition line between phases B and C now from phase B, so that a fragmentation event occurs, we consider one single component network disconnecting into two equal clusters. As done before, imagine a situation two time steps before a possible fragmentation—network has two internally homogeneous components in opposite states. One of the nodes *i* is part of a bridge, i.e. it is connected to two nodes in the opposite cluster. Now, for the fragmentation to occur we need both of the links between the components to be rewired. The probability to rewire the first one is $\frac{1}{N}(2 / \mu )^q p (1-\frac{\epsilon }{2})+\frac{2}{N} (1 / \mu )^q p$. We have to select the node *i* (1/*N*) or one of its neighbours (2/*N*). An interaction must occur, what happens with probability $(a_j / \mu )^q$, where the number of active links is 2 for node *i* and 1 for each of its neighbours in the opposite cluster. Finally, a rewiring must occur with probability *p*. Additionally if node *i* was selected, it can not change its state due to noise ($1 - \frac{\epsilon }{2}$), otherwise fragmentation could not be achieved in two steps. The transition occurs, however, for very small values of noise and therefore we can approximate $1 - \frac{\epsilon }{2} \approx 1$. To rewire the second link we have to select one of the two nodes (2/*N*) connecting the link, an interaction must occur $(1 / \mu ^q)$, which must be a rewiring event (*p*). Therefore, the probability of losing the last link between the two clusters is $\frac{2}{N} (1 / \mu )^q p$. Finally, we obtain the probability of disconnecting two clusters sharing only two links:

9 $$\begin{aligned} P_d = \left[ \frac{1}{N} (2 / \mu )^q p + \frac{2}{N} (1 / \mu )^q p \right] \frac{2}{N} (1 / \mu )^q p. \end{aligned}$$

Between phases B and C a continuous fragmenting and reconnecting of the network is observed. We define the transition between the two phases when connection and fragmentation happens at such a rate that half of the time the system consists of two separate components and half of the time the network is connected. Therefore, at the transition line we expect $P_r = P_d$, which leads to the equation for the critical density of noise given in the main text (Eq. 1).

## Supplementary information

Supplementary Legends.

Supplementary Video 1.

Supplementary Video 2.

Supplementary Video 3.

Supplementary Video 4.
